# Enhancement of titanium surfaces using different acid solutions at room temperature to improve bone cell responses

**DOI:** 10.1016/j.jds.2024.06.011

**Published:** 2024-06-26

**Authors:** Thu Ya Linn, Eisner Salamanca, Chun-Yu Ho, Yi-Fan Wu, Hao-Chun Chiu, Wei-Jen Chang, Ying-Sui Sun

**Affiliations:** a School of Dentistry, College of Oral Medicine, Taipei Medical University, Taipei, Taiwan; b Division of Oral and Maxillofacial Surgery, Department of Dentistry, Wan Fang Hospital, Taipei Medical University, Taipei, Taiwan; c School of Oral Hygiene, College of Oral Medicine, Taipei Medical University, Taipei, Taiwan; d Department of Biomedical Engineering, Ming-Chuan University, Taoyuan, Taiwan; e Department of Chemical Engineering, KU Leuven, Heverlee, Belgium; f Dental Department, Shuang-Ho Hospital, Taipei Medical University, New Taipei, Taiwan; g School of Dental Technology, College of Oral Medicine, Taipei Medical University, Taipei, Taiwan

**Keywords:** Acid etching, Surface modification, Titanium, Dental implant, Biocompatibility

## Abstract

**Background/purpose:**

Early osseointegration of titanium (Ti) dental implants relies on the surface topography. Surface modification of Ti seeks to enhance bone regeneration around implants. Acid etching is the simple, less technique sensitive and cost-effective technique for surface treatment. The purpose of this study was to elucidate the simplified acid etching technique at room temperature lies in its capacity to enhance both physical properties and biological reactions relevant to bone.

**Materials and methods:**

Utilizing sulfuric acid (H_2_SO_4_) and hydrochloric acid (HCl), five distinct acid solutions were prepared, and the acid etching process was executed at five different time points at room temperature. The surface characterization of nanoscale modified titanium disks encompassed surface characteristics analysis, wettability and roughness tests. The biocompatibility evaluation involves tests that assess cell attachment, proliferation, alkaline phosphatase activity (ALP), and mineralization.

**Results:**

The surface modified by HCl exhibited the most significant alterations, characterized by an elevated roughness value and reduced hydrophilicity properties. The surface treated with a mixture of HCl and H_2_SO_4_ for 24 h (TH5) displayed a hydrophilic surface and high surface energy. Acid etched surfaces showed the greater cell attachment with long pseudopodia. The cell proliferation rate and ALP reaction rate of TH5 is the highest at day 7. Cells mineralization of Ti surface treated with 37% HCl for 24 h (TC5) shows the lowest and TH5 shows the greatest on day 21.

**Conclusion:**

The proposed acid etching at room temperature utilizing a combination of H_2_SO_4_ and HCl demonstrated improved physical properties while fostering favorable biological responses.

## Introduction

Titanium (Ti) dental implants are widely-used for tooth replacements because of better osseointegration leading to long-term success rates. Key factors influencing early-stage osseointegration include the implant's surface roughness and chemical composition that affect the bone-and-implant interaction.^1^ The absence of biological responsiveness and constrained durability in Ti may raise compatibility issues with the bone, thereby increasing the likelihood of implant failure.^2^ Implant failure would require secondary surgical procedures that incurs substantial expenses, and adversely impacts the individual's quality of life.^3^ Hence, research interests are expanding on Ti surface modification, aiming to accelerate bone regeneration around dental implants, mitigate unwarranted peri-implant inflammation, and enhance antibacterial attributes of implant materials.^4^

In dental implants, Ti surface modification achieves the required surface topography through mechanical and chemical methods, such as acid etching (AE), anodic oxidation, or laser blasting.^5^^,^^6^ Many of these techniques exhibit complexity, sensitivity to specific techniques, high cost, and significant energy consumption. AE has benefits compared to other techniques because of its simplicity and cost-effectiveness. AE uses various acid solutions resulting in significant physical changes to attain a more bioactive surface of Ti implants.^7^ Different acid solutions result in diverse surface morphologies and biological responses. Thus, the choice of acid solution is crucial in achieving a better bioactive surface.

Prior studies have documented that Ti surface is sandblasted and acid etched with sulfuric acid (H_2_SO_4_) induces rapid alterations in surface properties and elicits more potent biological responses compared to hydrochloric acid (HCl).^8^^,^^9^ Ti surface treated with hydrofluoric acid (HF) or combination of concentrated HCl and H_2_SO_4_ at elevated temperatures exhibited microrough surface structure that facilitated sustained success in long-term.^10^^,^^11^ Utilizing dual AE with HF and HCl/H_2_SO_4_ revealed micro-rough surface which enhanced resistance to reverse torque removal and improved osseointegration.^5^ Exclusively, AE can eliminate contaminants and generate pits on the Ti surface positively impacting osseointegration by facilitating bone interlocking.^12^ Many previous studies provided the significance of acid solution concentration and types, reaction temperature, and etching duration, as they significantly impact the final outcomes.^13^ Nonetheless, the use of AE without sandblasting on implant surfaces remains an area lacking sufficient exploration for roughening implants via this method. On the other hand, numerous commercial dental implants are manufactured at elevated temperature, showcasing optimal biological results.^14^ Since the simplicity and reduced hazards associated with room temperature AE, further literature is needed to validate its efficacy in enhancing physical properties and biological reactions related to bone.

The objective of this study was to evaluate the surface characterization changes and biological cellular responses to various novel acid-etched surfaces based on their different precursor acid solutions.

## Materials and methods

### Sample preparation

**Figure 1:**
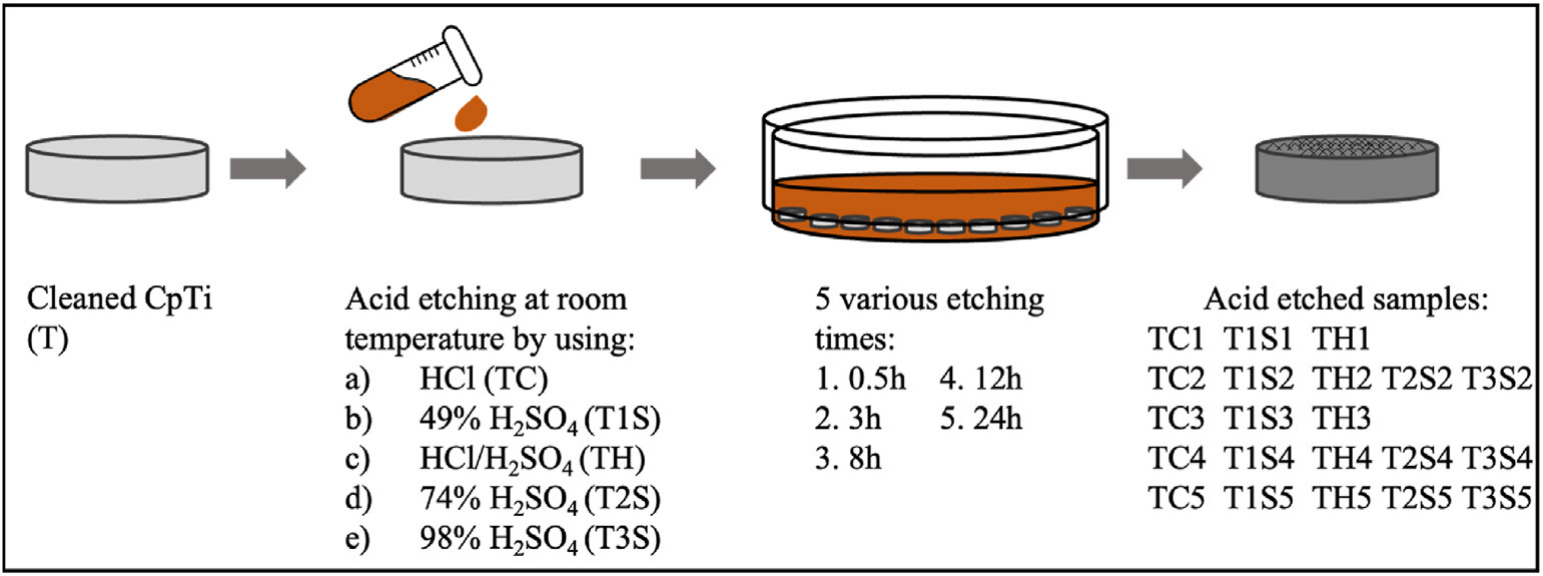
Process of acid etching on the titanium surface at room temperature.

Commercially pure Ti disks, grade IV (10 mm × 1 mm) were used (BioTech One Inc., Taipei, Taiwan). Samples were cleaned in 75% alcohol and double-distilled water (ddH_2_O), which is denoted as control group (T). The precursor solutions of HCl (Fluka™, Honeywell International Inc., Muskegon, MI, USA) and H_2_SO_4_ (J.T. Baker™, Avantor Inc., Phillipsburg, NJ, USA) were purchased from manufacturers. Ti disks were treated at room temperature with 37% HCl (TC), 48% H_2_SO_4_ (**T1S**), and a mixture (1:1) of 5.80 mol/L HCl and 8.96 mol/L H_2_SO4 (TH) for 5 different time points. Also, Ti disks were treated with 74% H_2_SO_4_ (T2S) and 98% H_2_SO_4_ (T3S) at room temperature for 3 h, 12 h, and 24 h (Fig. 1). Specimens were then ultrasonically cleaned with ddH_2_O and prepared for analysis.

### Characterization of physical and chemical properties

Surface topography was analyzed with scanning electron microscopy (SEM), after gold-palladium coating (SU3500, Hitachi Ltd., Tokyo, Japan). Images were taken at three random spots with two points of magnifications. Elemental composition was observed by energy dispersive X-ray analysis (EDS) (EDS; XFlash**®**6–60, Bruker, Billerica, MA, US). X-ray diffraction (XRD) provides the crystalline material structure of the surface (Empyrean, Malvern panalytical Ltd., Malvern, UK). Analysis of surface chemical composition was carried out by X-ray photoelectron spectroscopy (XPS) (PHI500 VersaProbe, ULVAC- PHI Inc, Kanagawa, Japan). Fourier transform infrared spectroscopy (FTIR) is used for the detection of surface functional groups (FTIR; Thermo-Fisher Scientific, Waltham, MA, USA). Hydrophilicity of specimens was evaluated by water contact angle measurement (model 100SB, Sindatek Instruments Ltd., Taipei, Taiwan). Atomic force microscope (AFM) was used to measure the surface roughness (AFM 1000, Suzhou Flyingman Precision Instruments Ltd., Suzhou, China).

### Characterization of biological properties

#### Evaluation of cell attachment and cell proliferation

For cell attachment analysis, MG-63 cells (No.60279, BCRC, Hsinchu, Taiwan) were seeded with density of 1 × 10^4^ cells/disk. Cell attachment was verified after culturing for 3 h, 6 h, 12 h and 24 h. Samples were immersed in a mixture of 2% glutaraldehyde, 4% paraformaldehyde, and cacodylate buffer solution for 24 h at 4 °C. Specimens were dehydrated in the ethanol solution in ascending order (10%–100%) and analyzed with SEM. For cell proliferation analysis, cells were seeded with a density of 5 × 10^3^ cells/disk. The proliferation was calculated by alamarBlue assay for 1, 3, 5, and 7 days (Biosource International, Lewisville, TX, USA) using an absorbance-based plate reader (Spectramax iD3, Molecular Devices, LLC., San Jose, CA, USA).

#### Analysis of cell differentiation and cell mineralization

MG-63 cells were seeded with same density of 5 × 10^3^ cells/disk for both analyses. Cell differentiation was evaluated by using colorimetric alkaline phosphatase (ALP) assay kit (ab83369, Abcam, Cambridgeshire, UK) at 7, 10, and 14 days. According to the manufacturer's instructions, 70 ml of cell lysate from each specimen and 50 ml of reagent were added to the 96-well plate. The absorbance was detected using absorbance-based plate reader at 405 nm. Cells mineralization analysis was performed at 10, 14, and 21 days. Cells were fixed with 70% alcohol and 2% alizarin red S (ARS) solution (Sigma-Aldrich, St Louis, MO, USA) was added in each well, incubated for 30 min at room temperature. Stained disks were photographed with upright microscope (Eclipse Ci-L Plus, Nikon, Tokyo, Japan) and HDMI microscope. The quantification of ARS staining was carried out by absorbance-based plate reader at 540 nm.

### Statistics analysis

The data from the experimental analyses were described using means and standard deviations. One-way ANOVA and Tukey's multiple comparisons were performed to compare multiple groups. All statistical analyses were carried out using SPSS statistical software (IBM SPSS Statistics, IBM Corp, Armonk, NY, USA). Values of *P* < 0.05 were considered statistically significant.

## Results

### Characterization of acid etched titanium surface

**Figure 2:**
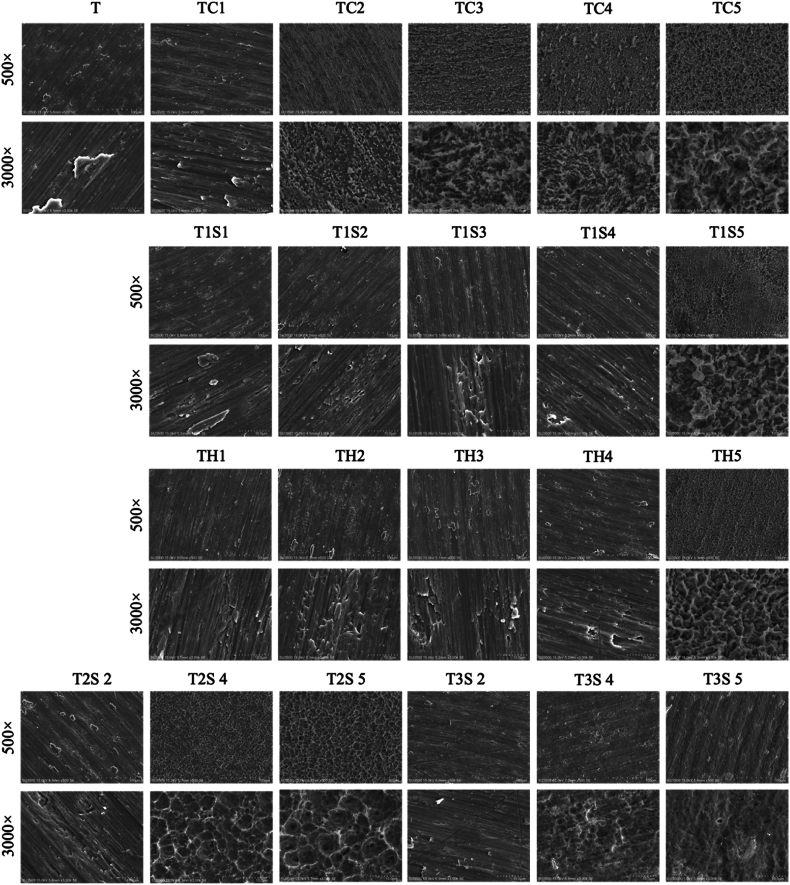
SEM images of the acid etched samples for characterization of surfaces. T: untreated titanium surface, TC: titanium treated with hydrochloric acid, T1S: titanium treated with 48% sulfuric acid, TH: titanium treated with the mixture of hydrochloric and sulfuric acid, T2S: titanium treated with 74% sulfuric acid, T3S: titanium treated with 98% sulfuric acid, SEM: scanning electron microscopy.

**Figure 3:**
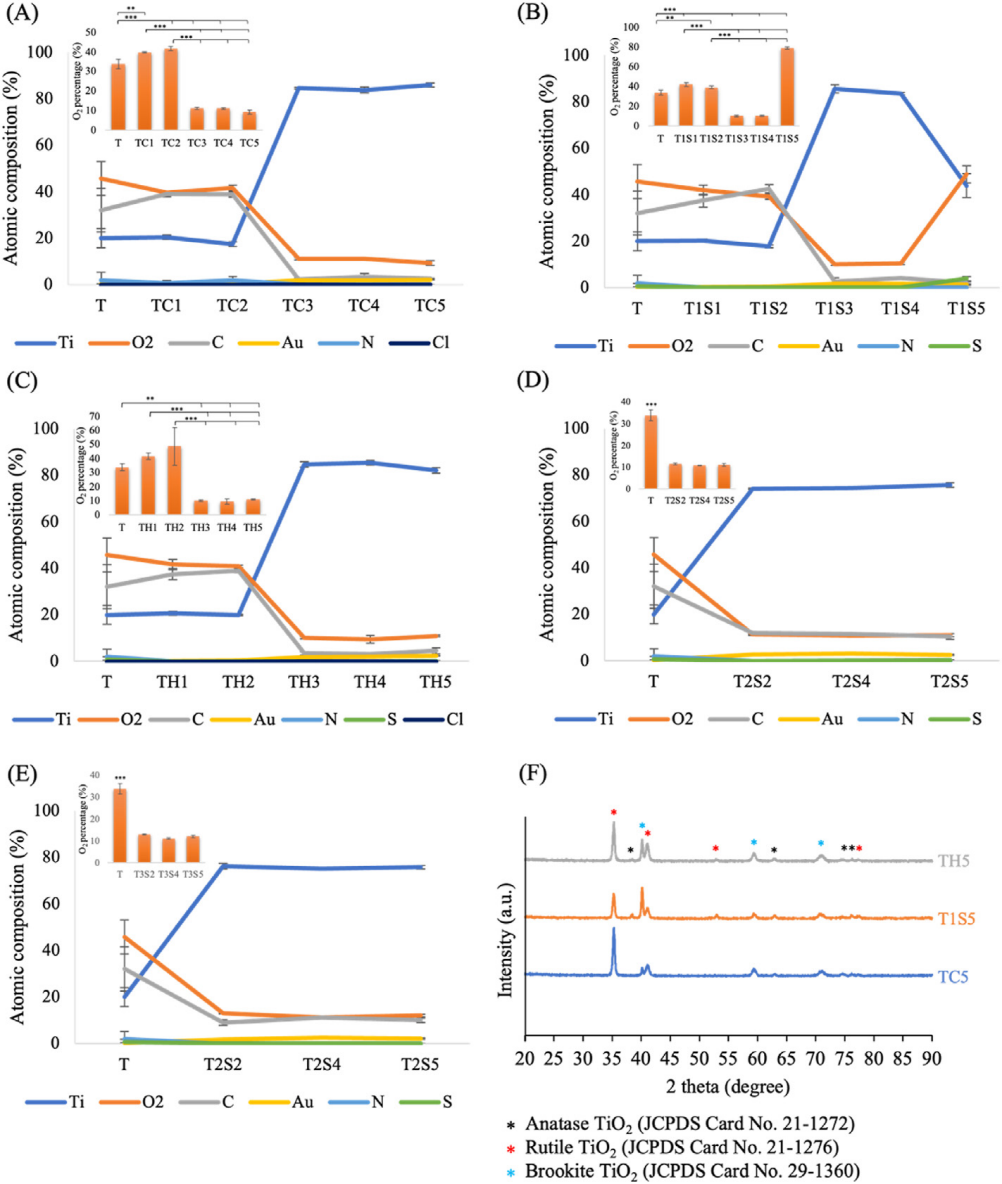
Illustration of energy dispersive X-ray analysis of acid etched samples: treated by (A) Hydrochloric acid, (B) Sulfuric acid, (C) Mixture of hydrochloric & sulfuric acid, (D) 74% Sulfuric acid and (E) 98% Sulfuric acid. (F) X-ray diffraction analysis of 24hr acid etched samples. TC5: titanium treated with hydrochloric acid for 24 h, T1S5: titanium treated with 48% sulfuric acid for 24 h, TH5: titanium treated with the mixture of hydrochloric and sulfuric acid for 24 h.

Generally, the Ti surface color changed to black or grey after 24 h of AE, with the TC group's color darkening from 3 to 24 h (Supplementary Fig. 1). SEM images (Fig. 2) showed prominent pits and craters on the TC5 group's surface. Using H_2_SO_4_ and HCl/H_2_SO_4_ mixtures did not significantly alter the surface topography in the T1S1, T1S2, TH1, and TH2 groups. In EDS analysis, the content of Ti was elevated (Table 1) when the treatment duration was increased in TC group which showed in Fig. 3, TC5 was the highest (85.95% ± 0.87). The rest of the elements such as oxygen, carbon, nitrogen, is reduced while the etched duration is increased. The titanium oxide is important in the biological responses of the newly developed Ti surface. The O_2_ percentage decreased when the treatment duration was longer in all groups (Fig. 3). However, O_2_ percentage of T1S surfaces increased at 24 h treatment time.

**Table 1:** Composition of elements on acid etched samples in five different etching time points by EDS (Atom %). EDS: energy dispersive X-ray analysis.

(mean ± SD)	Ti	O_2_	C	Au	N	S	Cl
T	19.98 ± 4.1	45.72 ± 7.33	32.08 ± 9.44	0.29 ± 0.09	1.93 ± 3.34	0.67 ± 1.15	0
TC1	20.45 ± 0.85	39.63 ± 0.25	38.96 ± 1.13	0.27 ± 0.05	0.67 ± 1.17	–	0
TC2	17.41 ± 0.97	41.65 ± 1.11	38.82 ± 1.15	0.29 ± 0.01	1.82 ± 1.65	–	0
TC3	84.59 ± 0.41	11.18 ± 0.47	2.32 ± 0.68	1.89 ± 0.28	0	–	0.02 ± 0.03
TC4	83.79 ± 1.32	11.05 ± 0.37	3.3 ± 1.49	1.86 ± 0.11	0	–	0
TC5	85.95 ± 0.87	9.28 ± 1	2.63 ± 0.12	2.11 ± 0.16	0	–	0.03 ± 0.03
T1S1	20.21 ± 0.59	41.99 ± 2.08	37.48 ± 2.73	0.28 ± 0.01	0.05 ± 0.09	0	–
T1S2	17.81 ± 0.58	39.23 ± 1.26	42.60 ± 1.71	0.35 ± 0.02	0.003 ± 0.006	0	–
T1S3	85.53 ± 1.78	10.11 ± 0.58	2.67 ± 1.47	1.68 ± 0.15	0	0.01 ± 0.02	–
T1S4	83.6 ± 0.49	10.49 ± 0.52	4.04 ± 0.18	1.82 ± 0.11	0	0.04 ± 0.05	–
T1S5	43.93 ± 5.22	48.74 ± 3.67	2.15 ± 0.48	1.32 ± 0.25	0	3.86 ± 1.0	–
TH1	20.63 ± 0.78	41.64 ± 2.23	37.51 ± 2.38	0.19 ± 0.05	0	0.03 ± 0.04	0
TH2	19.86 ± 0.30	44.90 ± 0.53	38.96 ± 0.79	0.29 ± 0.04	0	0	0
TH3	84.78 ± 1.21	10.07 ± 0.51	3.42 ± 0.77	1.71 ± 0.28	0	0.01 ± 0.02	0.003 ± 0.006
TH4	85.4 ± 1.04	9.43 ± 1.71	3.09 ± 0.5	2.05 ± 0.28	0	0.02 ± 0.03	0.003 ± 0.006
TH5	82.12 ± 1.26	10.88 ± 0.37	4.5 ± 1.26	2.38 ± 0.13	0	0.11 ± 0.03	0
T2S2	73.98 ± 0.23	11.48 ± 0.45	11.86 ± 0.42	2.64 ± 0.17	0	0.03 ± 0.03	–
T2S4	74.44 ± 0.05	10.78 ± 0.1	11.58 ± 0.15	3.11 ± 0.02	0	0.08 ± 0.02	–
T2S5	75.71 ± 1.06	11.03 ± 0.65	10.44 ± 1.21	2.54 ± 0.31	0	0.27 ± 0.12	–
T3S2	76.16 ± 1.18	13.01 ± 0.16	8.98 ± 1.21	1.82 ± 0.23	0	0.03 ± 0.02	–
T3S4	75.1 ± 0.12	11.13 ± 0.41	11.11 ± 0.21	2.60 ± 0.34	0	0.03 ± 0.03	–
T3S5	75.75 ± 0.68	12.08 ± 0.53	10.02 ± 0.92	2.09 ± 0.29	0	0.05 ± 0.02	–

T: untreated titanium surface, TC: titanium treated with hydrochloric acid, T1S: titanium treated with 48% sulfuric acid, TH: titanium treated with the mixture of hydrochloric and sulfuric acid, T2S: titanium treated with 74% sulfuric acid, T3S: titanium treated with 98% sulfuric acid.

**Figure 4:**
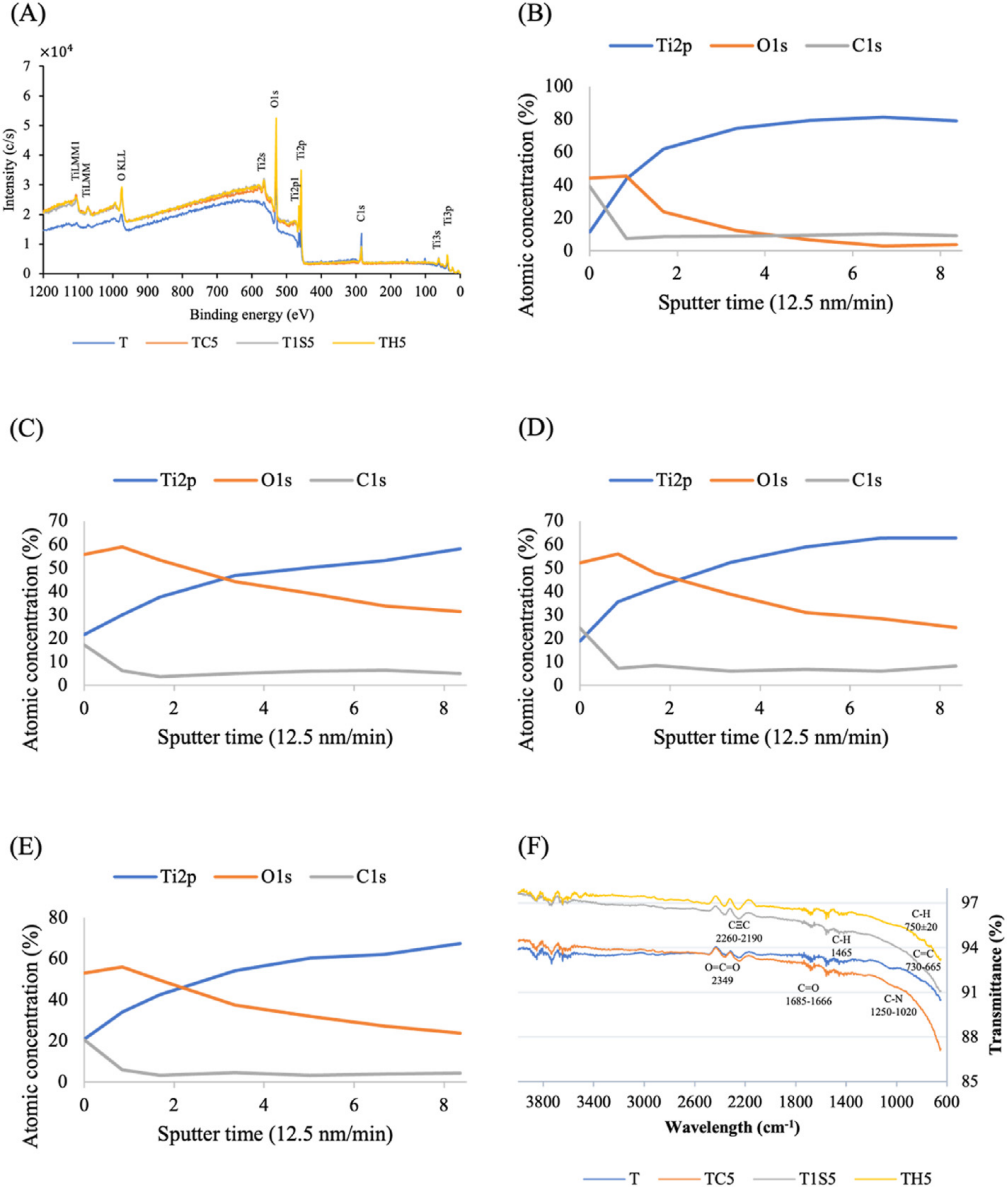
(A) XPS full spectra of acid etched group. Elemental depth profiles of (B) control titanium group (T), (C) titanium treated with hydrochloric acid for 24 h (TC5), (D) titanium treated with 48% sulfuric acid for 24 h (T1S5), and (E) titanium treated with the mixture of hydrochloric and sulfuric acid for 24 h (TH5). (F) Fourier-transform infrared spectroscopy analysis of acid etched samples. XPS: X-ray photoelectron spectroscopy.

XRD pattern of Ti surface treated with the different acid solutions shows in Fig. 3 (F). The phase structure of the different acid etched samples was examined in the JCPDS card: anatase TiO_2_ (Card No. 21–1272), rutile TiO_2_ (Card No. 21–1276) and brookite TiO_2_ (Card No. 29–1360). After AE, the O1s and Ti2p peaks of XPS spectra are significantly higher in all samples but the peak of C1s at the 285eV becomes lower, Fig. 4 (A). In elemental depth profile, O1was deeply seeded in larger concentration in the acid-etched samples, Fig. 4 (B-E). Ti2p proportions are gradually increased with the replacement of O1s.

FTIR spectrum of the acid etched sample at the 2253 cm^−1^ indicates CΞC stretching which indicated alkyne compound class, shown in Fig. 4 (F). TH5 showed the bending at the wavelength of 770 cm^−1^ and 725 cm^−1^ that represented the C–H and C=C groups respectively.

**Figure 5:**
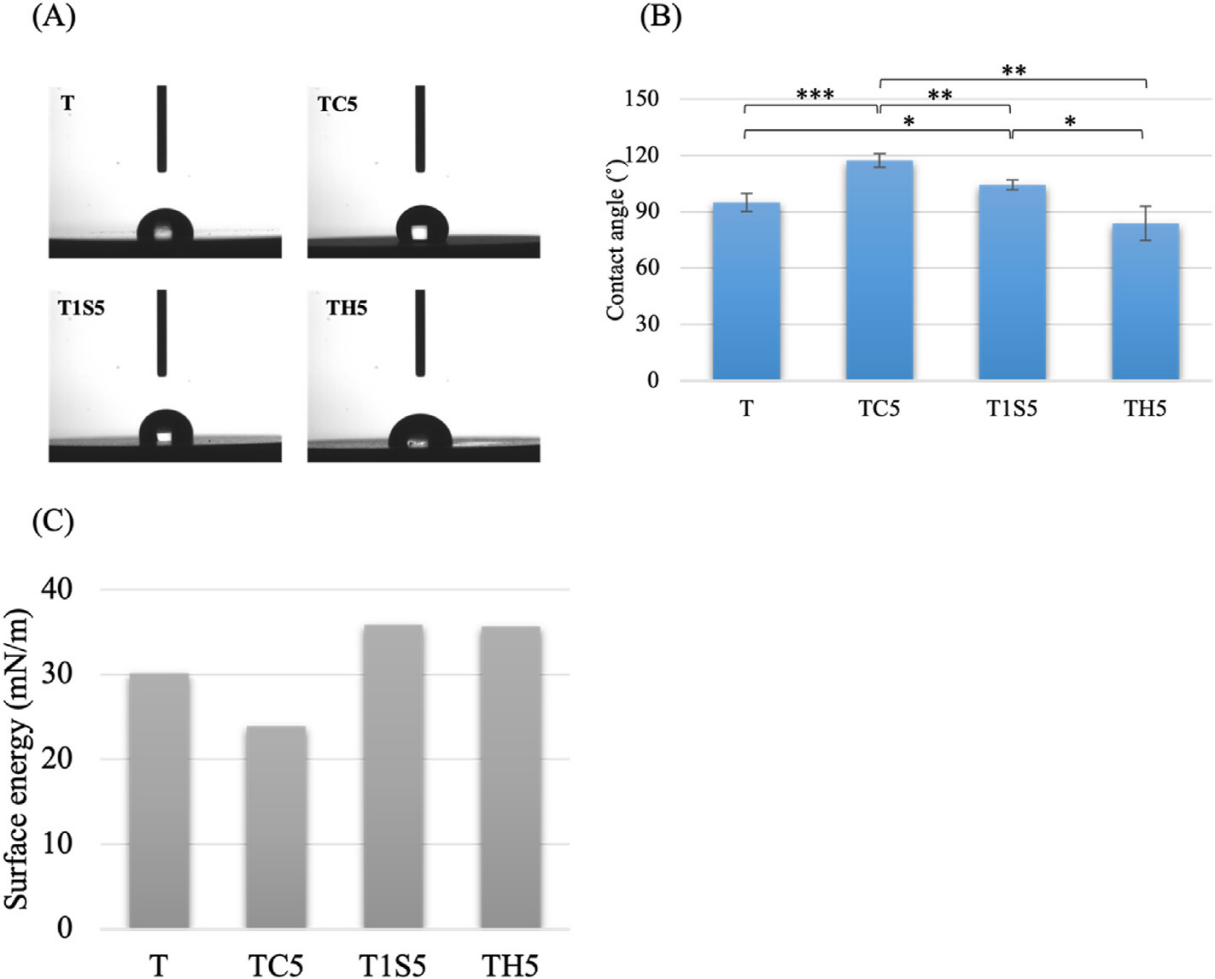
Water contact angle measurement of acid etched samples (A) real time image, (B) hydrophilicity properties, and (C) surface energy. T: untreated titanium surface, TC5: titanium treated with hydrochloric acid for 24 h, T1S5: titanium treated with 48% sulfuric acid for 24 h, TH5: titanium treated with the mixture of hydrochloric and sulfuric acid for 24 h. (∗*P* < 0.05, ∗∗*P* < 0.01, ∗∗∗*P* < 0.001).

**Figure 6:**
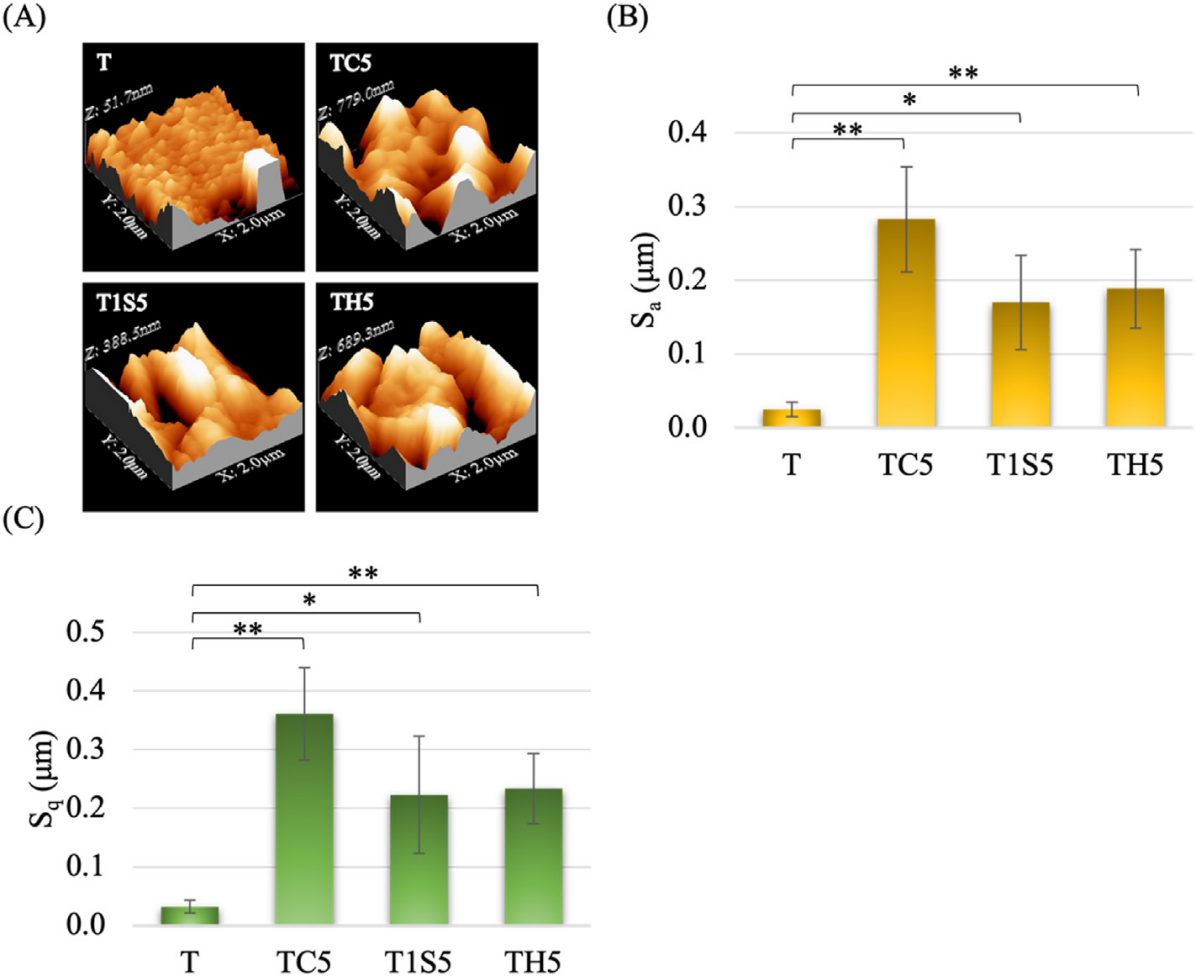
(A) Surface roughness analysis of acid etched samples in 3D images. Illustrations of (B) Sa value and (C) Sq value. T: untreated titanium surface, TC5: titanium treated with hydrochloric acid for 24 h, T1S5: titanium treated with 48% sulfuric acid for 24 h, TH5: titanium treated with the mixture of hydrochloric and sulfuric acid for 24 h. (∗*P* < 0.05, ∗∗*P* < 0.01, ∗∗∗*P* < 0.001).

Water contact angle measurement of TC5 and T1S5 groups are 117.25° ± 3.48 and 104.31° ± 2.72 respectively, Fig. 5. TH5 is significantly lower than the TC5 (*P* < 0.05) and T1S5 group (*P* < 0.01). Moreover, the surface energy of samples was calculated with Owens–Wendt method. Arithmetic mean height (S_a_) value of T group is the lowest (0.024 ± 0.01 μm) and that of TC5 group is the highest (0.284 ± 0.07 μm), which illustrated in Fig. 6.

### Evaluation of cell attachment and cell proliferation capacity

**Figure 7:**
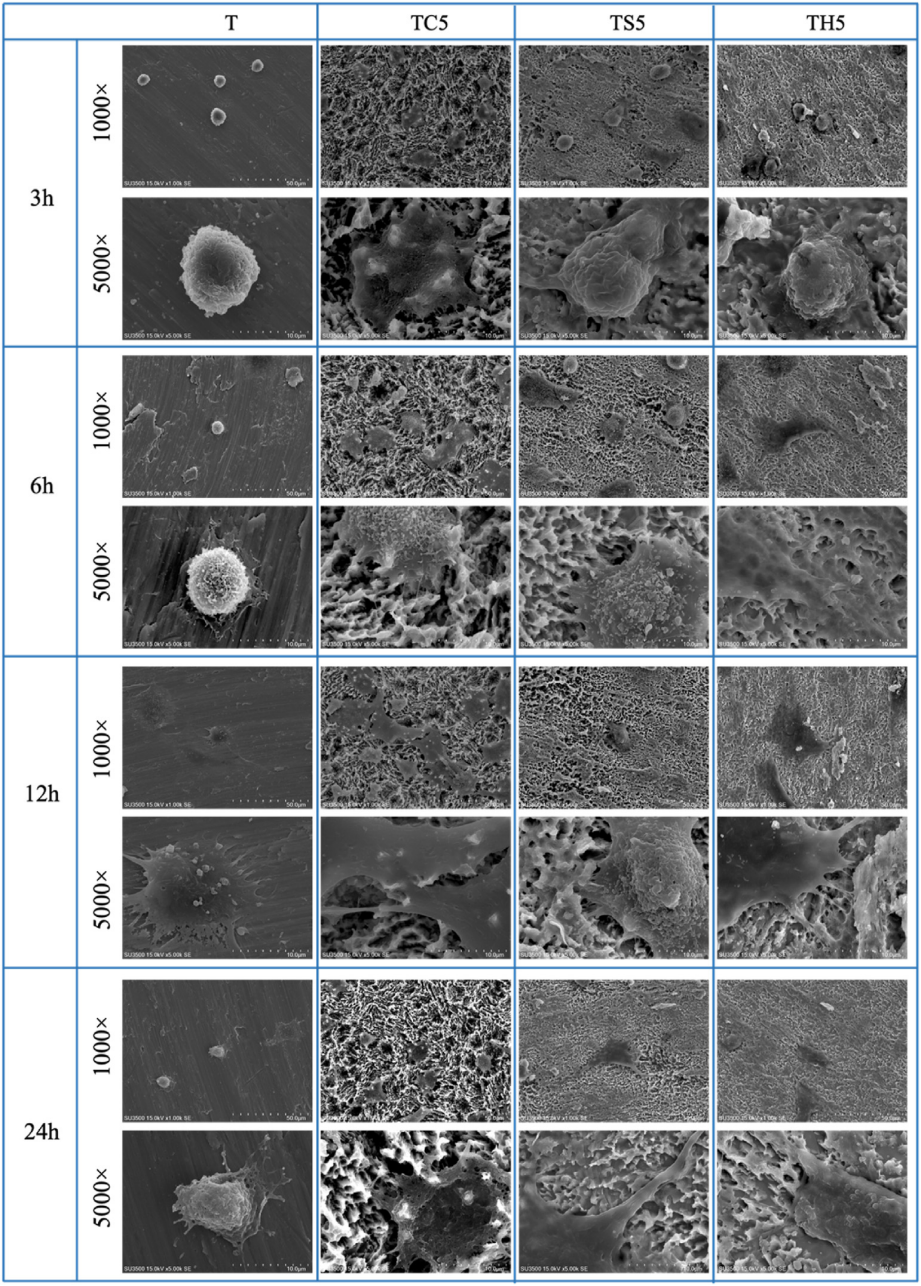
MG 63 cells attachment analysis by using SEM images at 3 h, 6 h, 12 h and 24 h of cells proliferation. T: untreated titanium surface, TC5: titanium treated with hydrochloric acid for 24 h, T1S5: titanium treated with 48% sulfuric acid for 24 h, TH5: titanium treated with the mixture of hydrochloric and sulfuric acid for 24 h, SEM: scanning electron microscopy.

Cells are well adapted to the crater like surfaces of acid etched samples and cells size were prominently larger, Fig. 7. Some cells are flattened at 12 h, and long spindle shaped pseudopodia are connected at 24 h.

**Figure 8:**
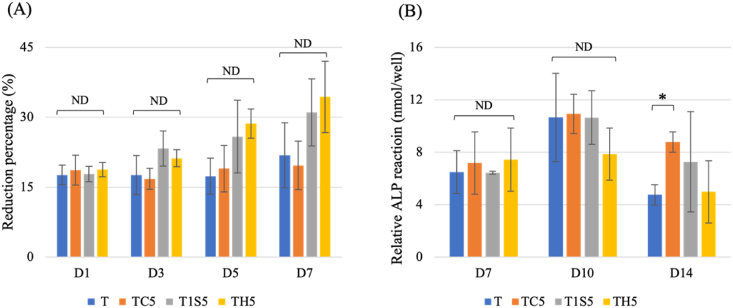
MG 63 cells (A) proliferation analysis by using almar Blue assay, and (B) cells differentiation analysis by using ALP assay. T: untreated titanium surface, TC5: titanium treated with hydrochloric acid for 24 h, T1S5: titanium treated with 48% sulfuric acid for 24 h, TH5: titanium treated with the mixture of hydrochloric and sulfuric acid for 24 h. (ND: no difference statistically, ∗*P* < 0.05).

The proliferation reduction percentage on the day 5 and 7 shows the same scenario, TH5 groups had the highest percentage (Fig. 8 (A)). However, there is no statistical significance difference between groups in various culture time.

### Analysis of cell differentiation and cell mineralization

Cell differentiation of the acid etched samples is shown in Fig. 8 (B). ALP activities on the modified surface of the acid etched groups showed similar reaction between groups and there is no significant difference was detected on day 7 and day 10.

**Figure 9:**
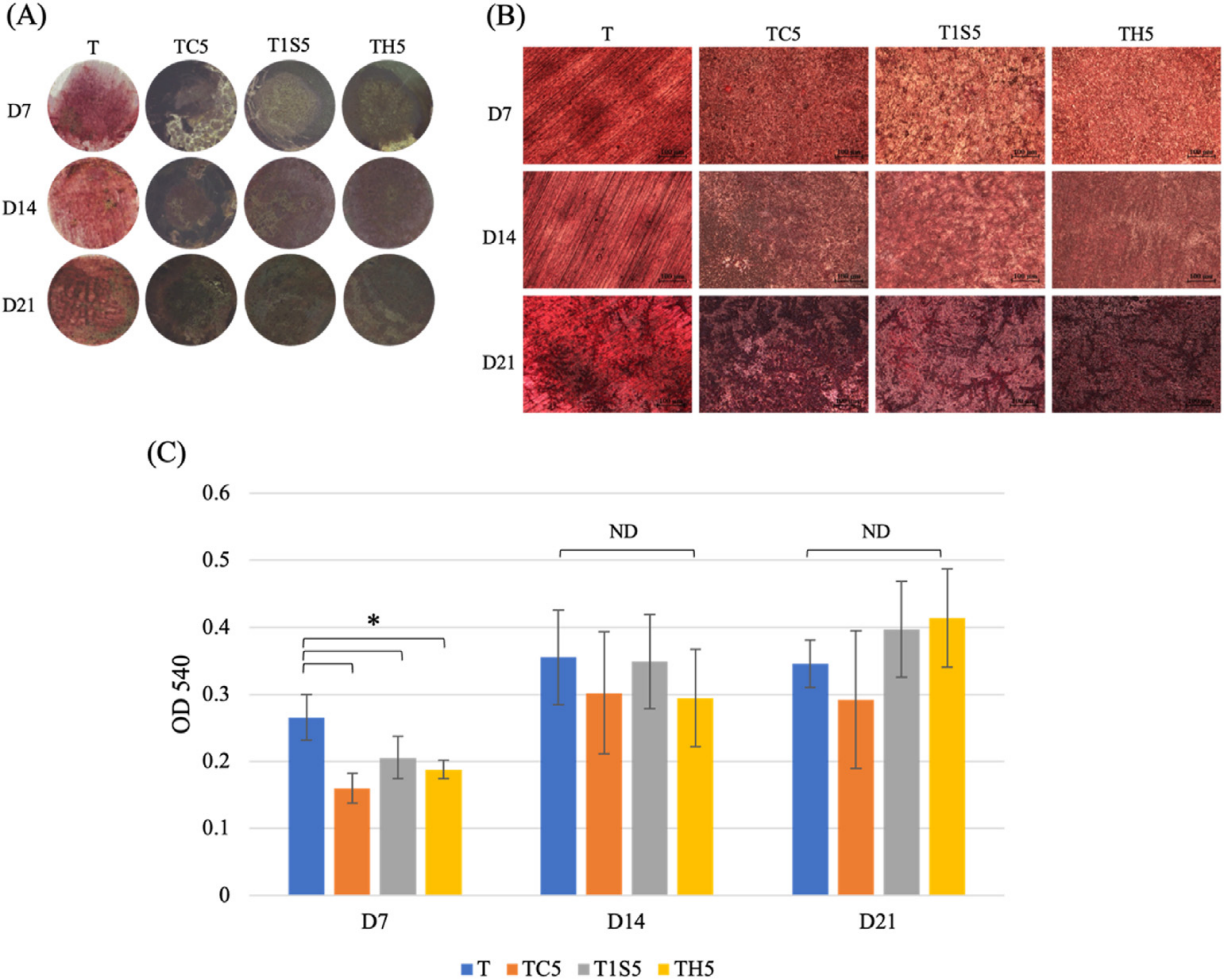
Cell mineralization of MG 63 cells (A) HDMI microscopic image, (B) upright microscopic image (10 × magnification), and (C) quantitative analysis at Day 7, Day 14 and Day 21. T: untreated titanium surface, TC5: titanium treated with hydrochloric acid for 24 h, T1S5: titanium treated with 48% sulfuric acid for 24 h, TH5: titanium treated with the mixture of hydrochloric and sulfuric acid for 24 h. (ND: no difference statistically, ∗*P* < 0.05).

The TH5 and T1S5 samples showed the greater calcification at day 21 (Fig. 9). The quantification optical density (OD) value of T group is significantly higher than other groups at day 7 but there is no difference between control and modified surface at day 14 and day 21.

## Discussion

Titanium dental implants are widely used to replace missing teeth. Implant success is evaluated based on mobility, peri-implantitis, and other complications.^15^ Surface characteristics are crucial for early osseointegration, influenced by osteoblastic cells. Subtractive method like acid etching increase implant surface area, enhancing early bone integration and implant stability.^13^ In this study, the effects of AE on the Ti surface at room temperature using various acid solutions are evaluated, comparing their physical and biological properties.

Ti surface color altered after AE. TC began changing from shiny to dull gray after 3 h, with darker colors at longer durations but TH became dull at 12 h and dark gray at 24 h. In agreement with the previous study,^14^ Ti surface etching with H_2_SO_4_ showed a lower response rate in surface changes when compared with other etching solutions. With increased etching duration, TC surfaces transformed micropits and pores like bone tissue in this study. TH5 also exhibited crater with pores. Micropits in T2S5 became larger and more uniformly distributed. The reaction rate for surface roughening with H_2_SO_4_ at room temperature was found to be lower.^6^ Notably, T1S5 only exhibited surface changes in this study. As micropits play a crucial role in the osseointegration,^16^ TH5 featured irregularly arranged 40 μm large valleys with 5 μm micropits. Therefore, the experiment at room temperature indicates that etching time significantly influences the acid reaction. Among the samples examined in this study, TC5, T1S5, and TH5 demonstrated the most substantial surface alterations following AE.

The reaction of sulfuric acid is faster depending on the concentration at which the elemental composition changes after 3 h in highly concentrated groups. TiO_2_ layer on the acid-etching sample can encourage the apposition of Ca and P ions from the surrounding environment to enhance osseointegration. Moreover, C is one of the elements found on the surface of a failed Ti implant.^17^ Thus, reducing C% benefits osteogenic bone cell activities. SEM and EDS data showed that TC5, T1S5, and TH5 had the greatest surface modification after AE, warranting further analysis.

Rutile TiO_2_ is the most frequently occurring in the TC5, T1S5, and TH5 because it is the most stable TiO_2_ in the strong AE process.^18^ The major peak at 41.36, which is also indexed to the rutile TiO_2_, was found to be the highest in the TH5 group. When surface topology showed well-defined pit and crater-like structures, the formation of rutile TiO2 structures was also more prominent. Elements on the surface, such as C and N composition, are relatively larger in the atmosphere. After applying surface treatment, the composition of C and N reduces nearly by half, with an increase in the O percentage.^19^ The change of elemental compositions aligns with the results of EDS analysis. With longer etching durations, the concentration peaks of Ti and O_2_ become intense, while that of C weakens.

Surface wettability is primarily influenced by chemical composition and topography. Increased roughness after AE made the surface hydrophobic.^20^ The contact angle and roughness of TC5 are significantly greater than other samples. TH5, with contact angle of 83.9°, is significantly lower due to the presence of alkene functional group. In a previous study,^8^ the arithmetic value of samples using mixture of HCl and H_2_SO_4_ at 25 °C was 0.22 ± 0.02 μm, which is similar to the Sa value of TH5 in the current study. Comparing to the commercial dental implant, the roughness of the BIOMET 3i implant is 0.79 μm, which used a dual acid-etching technique.^19^ The control Ti surface has lower roughness, but the increase after AE is significant across all acid-etched groups. Moreover, no pretreatment like sandblasting was done, resulting in smaller roughness values compared to previous studies.

Cells on the acid-etched samples appeared flattened with longer pseudopodia extensions as the culture time increased. Acid-etched samples provided an environment conducive to cell extension, forming spindle-like pseudopodia. Kieswetter et al. reported that roughened surfaces promoted cell extension, resulting thin and elongated morphology.^21^ Hence, TH5 manifested higher presence of lamellipodia, whereas T1S5 resulted in greater prevalence of filopodia. Moreover, TH5 and T1S5 exhibited higher cell proliferation rate compared to others. A separate study also revealed similar conditions in cell proliferation between acid-etched and unetched group.^22^ ALP activity of acid-etched surfaces in this study peaked at day 10, followed by a decline in activities at day 14. The reaction of modified surface is significantly higher than unmodified surface. Nonetheless, the mineralization of cells on the acid-etched surface exhibited a progressive increase, particularly within the T1S5 and TH5. By day 14, the TH5 surface manifested a reduced calcification area compared to the other acid-etched surfaces, aligning with the observed ALP activity reaction. The quantified absorbance of ARS staining for TH5 surpassed that of the other groups at day 21. Since the TH5 surface had the more hydrophilic properties, the biological reaction might be greatly influenced by the wettability properties. The present study summarized that the newly developed acid-etched surface promoted MG-63 cell responses in cell proliferation analysis, especially on day 7. Cell mineralization of MG-63 cells on the acid-etched surface was highest on day 21. The surface treated with a mixture of HCl and H_2_SO_4_ exhibited the most promising results in cell responses. The physical characteristics of TH5 supported the biological cell responses. The surface roughness of TH5 was lower than that of other samples. Moreover, wettability properties and surface chemical composition also influenced the MG-63 cell responses on titanium surfaces acid-etched at room temperature. Consequently, the utilization of a mixed acid solution in the AE process may induce superior biological responses, such as enhanced cell proliferation and mineralization.

This study introduced a novel room temperature acid etching method using various acid solutions. Within the constraints of this study, the Ti modified surface did not exhibit notable improvement biological cell responses including cell attachment, proliferation, and mineralization. The modified surface needs further modifications to improve the biological responses. However, it suggests that simple AE method at room temperature using concentrated HCl or a mixture of HCl and H_2_SO_4_ induces significant changes of physical and chemical properties.

## Declaration of competing interest

The authors have declared that there are no competing interests.
