# Supplementary material for: Enhancement of titanium surfaces using different acid solutions at room temperature to improve bone cell responses
Source: J Dent Sci. 2024 Jun 26;20(1):373–83. doi: 10.1016/j.jds.2024.06.011 (PMC11763216; doi:10.1016/j.jds.2024.06.011)

**Supplementary figure**

**Supplementary Figure 1**. Optical images of the different groups after acid etching process. TC: titanium treated with hydrochloric acid, T1S: titanium treated with 48% sulfuric acid, TH: titanium treated with the mixture of hydrochloric and sulfuric acid.


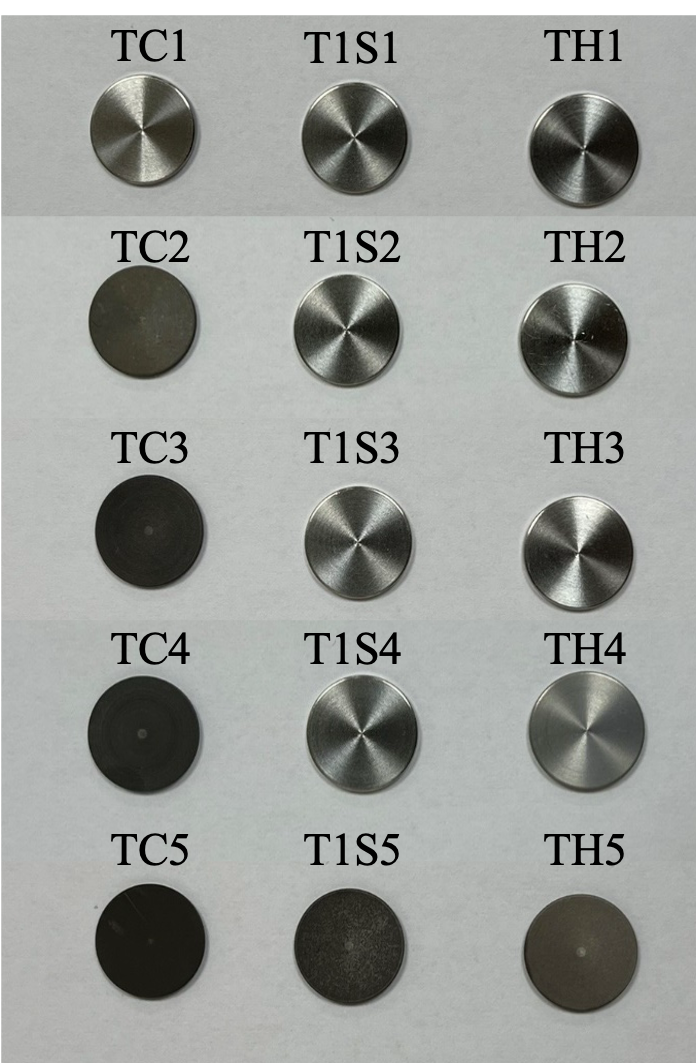

Supplement: Multimedia component 1 [file mmc1.docx]
